# An Internal Limiting Membrane Plug and Gas Endotamponade for Recurrent or Persistent Macular Hole

**DOI:** 10.1155/2019/6051724

**Published:** 2019-03-07

**Authors:** Fabrizio Giansanti, Ruggero Tartaro, Tomaso Caporossi, Daniela Bacherini, Alfonso Savastano, Francesco Barca, Stanislao Rizzo

**Affiliations:** Dipartimento Neuro-muscoloscheletrico e Organi di Senso, Azienda Ospedaliera Universitaria di Careggi, Florence, Italy

## Abstract

**Introduction:**

Recurrent or persistent macular holes (MHs) are rare today due to the tendency to carefully peel the internal limiting membrane. Conversely, their treatment is still a challenge for a vitreoretinal surgeon.

**Materials and Methods:**

This is a retrospective, consecutive, and nonrandomized study of patients affected by recurrent or persistent MHs treated using small-gauge pars plana vitrectomy (25- or 23-gauge) and an autologous ILM plug, at the Eye Clinic of Azienda Ospedaliera Universitaria Careggi (Florence, Italy) between January 2016 and May 2018. We included 8 eyes of 8 patients in the study. Five patients had a recurrent MH while 3 had a persistent MH. The case series includes patients with myopic eyes and with large macular holes (>400 *μ*). Patients were followed up with ophthalmoscopic examinations and swept-source optical coherence tomography (SS-OCT).

**Results:**

The mean age of the patients was 74 years (±4.81 standard deviation (SD)), 3 patients were men and 5 women. The average axial length was 26.28 mm (±2.84 SD). Four patients had an AL ≧ 26 mm. The mean MH diameter was 436.5 (±49.82 SD). Average preoperative best-corrected visual acuity (BCVA) was 0.81 logMAR (±0.16 SD) and 20/125 Snellen. The ILM plug has been found integrated in the MH in all the follow-ups.

**Conclusion:**

In our study, an ILM autologous macular transplant was used successfully in 5 cases of macular hole recurrence and 3 cases of macular hole persistence. The anatomical success was achieved in all the cases; 4 patients improved their BCVA, and 4 patients maintained it. No macular alterations such as RPE or retinal atrophy/dystrophy were observed after 6 months.

## 1. Introduction

Via pars plana (VPP) vitrectomy surgery for macular holes (MHs) has a high success rate, with recent reports of primary closure rates of more than 90% [1, 2]. Treatment of primary macular holes is mainly performed with macular peeling [3–7] or with inverted internal limiting membrane (ILM) flap [4, 6, 8–12], if the patient has a large hole or has high myopia. Unfortunately, macular holes can be persistent if they remain open after surgery, or recurrent if they reopen after initial closure. Previous studies showed an incidence of MH persistence or reopening after initial closure between 4.8% and 9.2% [13–16]. This may be due to residual epiretinal traction, insufficient gas tamponade, poor compliance by the patient in keeping a prone position, or an unknown cause [17]. Recurrent or persistent MH treatment is still a challenge for a vitreoretinal surgeon.

A technique of autologous ILM plug transplantation has already been proposed [18, 19].

This work aims at reporting the anatomical and functional results in a series of patients affected by recurrent or persistent MH treated using an autologous ILM plug transplant.

## 2. Materials and Methods

This is a retrospective, consecutive, nonrandomized, and comparative study of patients affected by recurrent or persistent MHs treated using small-gauge pars plana vitrectomy (PPV) (25- or 23-gauge) and an autologous ILM plug at the Eye Clinic of Azienda Ospedaliera Universitaria Careggi (Florence, Italy) between January 2016 and May 2018. We included 8 eyes of 8 patients in the study (Table 1).

Five patients had a recurrent MH while 3 had a persistent MH.

All the patients were pseudophakic and had previously undergone pars plana vitrectomy with macular ILM peeling for a macular hole. The average time for reopening of the MH (5 eyes) was 15. 4 months.

The mean age of the patients was 74 years (±4.81 standard deviation (SD)); 3 patients were men and 5 women. The average axial length was 26.28 mm (±2.84 SD). Four patients had an AL ≧ 26 mm. The mean MH diameter was 436.5 (±49.82 SD).

Average preoperative best-corrected visual acuity (BCVA) was 0.81 logMAR (±0.16 SD) and 20/125 Snellen. The patients had previously undergone surgery in different centers (3 patients in our centre by 3 different surgeons and 5 patients in different Italian centers all by different surgeons). The study was approved by the Institutional Ethics Committee and complied with the Declaration of Helsinki. Written informed consent for participation was obtained from all patients. All the patients underwent a complete ophthalmologic examination, and optical coherence tomography (OCT) (SPECTRALIS; Heidelberg Engineering, Germany; RS300 Advance SD-OCT; Nidek Co, Ltd, Japan; and DRI OCT Triton OCT, Topcon, Japan) was conducted at the time of surgery and 1 month, 4 months, and 6 months later.

The macular hole minimum and maximum widths were measured using a foveal OCT scan, as described by Duker et al. [20]. Both widths are measured using the OCT calliper function, as a line drawn roughly parallel to the RPE.

Visual acuity was converted into logMAR to perform the statistical analysis.

### 2.1. Surgical Technique

A standard 3-port 23/25-gauge PPV was carried out (CONSTELLATION, Alcon Surgical, Fort Worth, TX). The choice of the calibre of the vitrector was made according to the axial length (AL) of the eye; the 23-gauge was used for eyes with an AL ≧ 28 mm. An ILM dye Brilliant Blue G (Brilliant Peel, Fluoron, Germany) or Membrane Blue Dual (DORC, the Netherlands) was injected onto the ILM withdrawing area to stain the ILM for approximately 30 seconds. All the residual epiretinal membranes (ERMs), which were present in 2 patients, were removed. The ILM was harvested in an area inside the vascular arcades, starting from the edge of the previously removed ILM, in a circular fashion for approximately 1 disk diameter, and inserted into the hole (see Video, Supplemental Digital Content 1). A balanced salt solution and air exchange was performed, and gas (C3F8 14% or SF6 20%) was injected at the end of surgery. The patients were subsequently kept in a face-down position overnight and were advised to take a prone position for 3 days after surgery. Topical therapy with tobramycin and dexamethasone drops was carried out after surgery for 30 days.

## 3. Results

**Figure 1:**
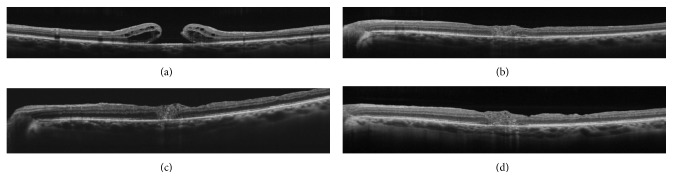
(a) Preoperative OCT that shows a recurrent macular hole (max. MH diameter 461). (b) 1 month after the operation, the plug is positioned into the closed macular hole. (c) 4 months after the operation. (d) 6 months after the operation. We can observe a partial reconstruction of the outer retinal layers.

Mean postoperative BCVA after 6 months was 0.68 logMAR (±0.14 SD) and 20/100 Snellen. The ILM plug was kept in place in all the cases and integrated in the MH to close it (see Figure 1). No eyes had postoperative macular degeneration such as RPE or retinal atrophy/dystrophy after 6 months. All 8 patients had successfully closed holes at 6 months and improved their BCVA (4 cases) or maintained it (4 cases). The myopic patients also had encouraging visual outcomes, which could be due to a preoperative absence of myopic patchy macular atrophy.

## 4. Discussion

The study shows 5 cases of recurrent MH and 3 cases of persistent MH who underwent VPP combined with autologous ILM plug transplant into the macular hole. The anatomical success was achieved in all the cases, and no cases of postoperative macular degeneration such as RPE or retinal atrophy/dystrophy were observed after 6 months.

This technique has already been described in the literature and has provided excellent results with success rates of 91–100% [21–26].

Concerning the inserting technique, the authors have tried to solve the problem of autologous ILM transplant instability within the MH in several ways. For example, some authors have used ocular viscoelastic devices (OVD) to stabilise the ILM flap [21, 23]. Morizane et al. [21] injected OVD over the ILM flap to position it in the hole and hold it in place. Dai et al. [23] instead used OVD to slightly lift the edge of the recurrent hole and prepare it to receive the ILM flap. Park et al. [22] used a drop of perfluoro-*n*-octane which, after stabilising the ILM flap inside the macular hole, was removed using a basic salt solution- (BSS-) air exchange.

We were able to insert the ILM flap without OVD or perfluoro-*n*-octane. The ILM flap floats into the vitreous cavity and can be easily lost during manipulation. We reduced the infusion to 10 mmHg to make the ILM plug easier to manoeuvre and avoid excessive flotation. The insertion of the plug must be carried out with great care to avoid damaging the macular edges. The thinness and the motility of the ILM plug allow good visualisation during the insertion manoeuvres, and the contact with the macular surface can be avoided (see supplemental digital content 1).

Risk factors for macular hole reopening include cataract surgery, intraoperative retinal tears, and cystoid macular oedema [27, 28]. High myopia is also a risk factor, and our series confirms that 4 out of 8 eyes (50%) had an axial length ≧ 26 mm.

All the patients had undergone phacoemulsification during the first vitrectomy; therefore, in our series, recurrence is independent of the lens removal. The pathophysiology causing the recurrence of the macular hole is not clear but probably relates to mechanical factors, inflammatory factors, or both [15, 29].

Previous studies reported that the average reopening time of a macular hole is between 12 and 15 months [30, 31]; although in 1 series, an average reopening time of 28 months was observed [32]. We found a result more similar to the literature, because in our series, the reopening time in the eyes with recurrent MHs was 14.6 months. Surgical technological advances, and especially small-gauge surgery and modern phacoemulsification, have led to decreased rates of macular hole reopening. In fact, the decreased postoperative inflammation that could cause a cystoid macular edema possibly reduces the macular hole reopening rates. Moreover, better surgical instrumentation and the assistance of vital dyes to identify ERMs and ILMs can help the surgeon to perform a more thorough peeling.

A longer reopening time is probably related to more consistent and improved ILM peeling. Better staining and visualisation methods and better surgical instruments are permitting more extensive and less traumatic macular peeling. Internal limiting membrane peeling is thought to reduce the recurrence rate of ERMs after macular surgery by eliminating a scaffold for cellular reproliferation [13, 33, 34]. In fact, ERMs may create a “spillover” effect from the gliosis that induced the macular hole to close after the first operation. The “spillover” effect derives from tangential traction that acts centrifugally and could counterbalance the centripetal forces that close a macular hole.

Histopathologic studies of ERMs associated with recurrent holes have shown Muller cells and astrocyte cells similar to those seen proliferating inside successfully closed holes [35, 36]. These cells could generate the extracellular matrix and therefore the centrifugal forces that reopen the macular hole.

In our series, ILM peeling had previously been performed in all cases, but in 2 cases, we had to remove, during the second operation, an ERM that had formed even in the presence of ILM peeling. Each eye that had additional ERM peeling during the second operation had successful macular hole closure. Moreover, residual ERM can increase foveal fluid, and if there is a noncomplete outer retinal layers postoperative reconstruction, we can have a failure in the RPE pump and then an increased risk of hole reopening due to an uncontrolled cystic accumulation.

Chakrabaarti and Roufail [37] used the same shorter-acting gas tamponade (SF6) without posturing instructions with reasonable success rates in traumatic MHs. We also used SF6, but we preferred to tell patients to stay face down to facilitate closure in these complex recurrent or persistent MHs cases. Gases are thought to increase the contact between the neurosensory retina and the RPE pump and therefore reduce cystic macular accumulation.

Other tamponading tools, such as “heavy” silicone oils, have been successfully proposed by Rizzo et al. [38–40] for the closure of persistent holes. In our series, we preferred to use a gas tamponade because we believe that a strong tamponading force in the first postoperative period is crucial for surgical success. Moreover, we do not have to remove the silicone oil, risking MH reopening. Injecting silicone oil may cause inflammation and emulsion; so, we only use it if multiple peripheral retinal tears could lead to the formation of a rhegmatogenous retinal detachment.

Chakrabarti et al. [41] only used sterile air for the postoperative tamponading of the MHs, but we have preferred gas in order to have a more prolonged effect.

The frequencies of bilateral MHs among the reopened cases in earlier studies were between 33% and 59% [15, 42, 43]. In our cases, similarly, 3 out of 8 patients had had a macular hole in the fellow eye. Patients with recurrent holes may tend to form ERMs and tractional forces in general that cause macular holes to open.

By analysing microstructural changes in the fovea using swept-source optical coherence tomography (SS-OCT) in eyes with large refractory MH following autologous ILM transplantation, Pires et al. [24] showed that the closure was associated with the prolonged proliferation of glial tissues in the fovea with fibrotic and depigmentation phenomena. Moreover, Ra and Lee [44] also described these phenomena.

In our study, conversely, we found good recovery of the outer retinal layers, (see Figure 1) which they had already been correlated positively with postoperative visual improvement [45]. Once the external limiting membrane (ELM) is damaged, the breaching of the seal between the neurosensory retina and the RPE pump causes fluid accumulation in the fovea with an elevation of the edges of the hole and, consequently, its progression and enlargement.

When the hole is successfully repaired, the migration of the glial cells bridges the hole and reestablishes the seal between the neurosensory retina and the RPE. This allows a fluid reduction in the cystic retina and therefore the macular hole closure [46].

The myopic patients had stable or improved visual outcomes, which may be due to a preoperative absence of myopic patchy macular atrophy. Moreover, we did not observe macular pigment epithelium atrophy in myopic patients after surgery. Concerning the hydrostatic theory of macular hole closure [46], the presence of the ILM plug could possibly reduce the entering of fluids into the retinal layers of the macula.

The limitations of this study are its retrospective nature, the small number of patients, and short-term follow-up. A prospective study and a more extensive series of cases using this technique could provide reliable and conclusive data on the efficacy of ILM autologous plugs and gas endotamponade in recurrent/persistent macular holes.

## 5. Conclusion

In our study, an ILM autologous macular transplant revealed its usefulness and safety in the cases of recurrent and persistent macular holes that had previously undergone surgery with vitrectomy and ILM macular peeling.

## Figures and Tables

**Table 1:** Preoperative findings and postoperative outcomes of the patients.

Patient	Age (years)	Maximum MH diameter (*μ*)	MH type	Axial length (mm)	Preoperative visual acuity (Snellen)	Preoperative visual acuity (logMAR)	Postoperative visual acuity (Snellen) after 6 months	Postoperative visual acuity (logMAR) after 6 months	Macular hole reopening time (months)	Macular hole in the fellow eye
1	75	461	Recurrent	24.7	20/200	1	20/200	1	18	Yes
2	69	385	Persistent	24.2	20/100	0.7	20/100	0.7	0	No
3	81	376	Recurrent	25.1	20/125	0.8	20/80	0.6	19	Yes
4	77	440	Recurrent	23.9	20/200	1	20/80	0.6	12	Yes
5	73	420	Recurrent	27.1	20/200	1	20/100	0.7	11	No
6	68	510	Persistent	28.4	20/100	0.7	20/100	0.7	0	No
7	79	485	Recurrent	27.8	20/80	0.6	20/63	0.5	17	No
8	70	415	Persistent	29.1	20/100	0.7	20/100	0.7	0	No

## Data Availability

The data used to support the findings of this study are included within the article.

## Supplementary Materials

Supplementary Materials Supplemental digital content 1: surgical video showing the ILM plug technique.
